# Novel* GDAP1* Mutation in a Vietnamese Family with Charcot-Marie-Tooth Disease

**DOI:** 10.1155/2019/7132494

**Published:** 2019-04-24

**Authors:** Phuong-Thao Mai, Dong-Truc Le, Tan-Trung Nguyen, Hoang-Linh Le Gia, Trung-Hieu Nguyen Le, Minh Le, Duc-Minh Do

**Affiliations:** ^1^Department of Physiology, Faculty of Medicine, University of Medicine and Pharmacy at Ho Chi Minh City, Vietnam; ^2^Center for Molecular Biomedicine, University of Medicine and Pharmacy at Ho Chi Minh City, Vietnam; ^3^Department of Biotechnology, Faculty of Chemical Engineering, Ho Chi Minh City University of Technology, VNU-HCM, Vietnam; ^4^Department of Neurology, Faculty of Medicine, University of Medicine and Pharmacy at Ho Chi Minh City, Vietnam; ^5^Department of Neurology, University Medicine Center at Ho Chi Minh City, Vietnam

## Abstract

**Background:**

Mutations of* GDAP1* gene cause autosomal dominant and autosomal recessive Charcot-Marie-Tooth (CMT) disease and over 80 different mutations have been identified so far. This study analyzed the clinical and genetic characteristics of a Vietnamese CMT family that was affected by a novel* GDAP1* mutation.

**Methods:**

We present three children of a family with progressive weakness, mild sensory loss, and absent tendon reflexes. Electrodiagnostic analyses displayed an axonal type of neuropathy in affected patients. Sequencing of* GDAP1* gene was requested for all members of the family.

**Results:**

All affected individuals manifested identical clinical symptoms of motor and sensory impairments within the first three years of life, and nerve conduction study indicated the axonal degeneration. A homozygous* GDAP1* variant (c.667_671dup) was found in the three affected children as recessive inheritance pattern. The mutation leads to a premature termination codon that shortens GDAP1 protein (p.Gln224Hisfs∗37). Further testing showed heterozygous c.667_671dup variant in the parents.

**Discussion:**

Our study expands the mutational spectrum of* GDAP1*-related CMT disease with the new and unreported* GDAP1* variant. Alterations in* GDAP1* gene should be evaluated as CMT causing variants in the Vietnamese population, predominantly axonal form of neuropathy in CMT disease.

## 1. Introduction

Charcot-Marie-Tooth disease (CMT) is one of the most common inherited peripheral neuropathies affecting motor and sensory neurons. More than 80 causative genes have been reported as autosomal dominant, autosomal recessive, and X-linked forms [1–3]. The disease features can be diverse even among those sharing the same mutation. Assessment of age at onset, key clinical findings (the dominance of motor or sensory, upper or lower limbs symptoms, and differentiating features), and family history are very useful in choosing a genetic testing strategy. The classical phenotype is usually characterized by early age at onset with slowly progressive weakness and atrophy of the distal muscles mostly in the lower limbs, foot deformities, walking impairment, areflexia, and mild sensory deficits [4]. Clinically, patients are classified into 3 groups based on the nerve conduction studies: a demyelinating (CMT1) with very slow motor nerve conduction velocity (MNCV), an axonal (CMT2) with normal or slight reduction of MNCV, and an intermediate phenotype. The demyelinating type occurs 2/3 of CMT cases with the duplication in* PMP22* gene, while the mutation spectrum in CMT2 is more diverse including variants in* MFN2*,* MPZ*,* GJB1*, and* GDAP1* and a large number of additional genes [5].

GDAP1 (Ganglioside-induced differentiation-associated-protein 1) is an integral membrane protein of the outer mitochondrial membrane with a ubiquitous tissue distribution but predominantly expressed in neurons. It is involved in many aspects of mitochondrial morphology and functioning, such as on the maintenance and regulation of normal functioning, structural integrity, and intracellular networking of the mitochondria [6].* GDAP1* is identified as a CMT-causative gene [7, 8] and its mutations have been shown to be responsible for an early-onset autosomal recessive demyelinating neuropathy [7], an axonal recessive form [8, 9], an intermediate recessive form [10], and a late-onset autosomal dominant axonal form [11–13].

*GDAP1* autosomal recessive inherited mutations cause a severe, early onset neuropathy often resulting in wheelchair-dependency in the second or third decade. Most of these patients develop unilateral or bilateral vocal cord paresis and diaphragmatic weakness in the latter stages of the disease [14]. It has been suggested that recessive mutations which cause truncating proteins develop a more severe phenotype, while missense mutations may be associated with a slightly milder progression [15]. Otherwise, autosomal dominant inherited mutations cause a much milder phenotype, characterized by adult onset, predominantly distal involvement, and slow progression [16, 17].

We herein report the clinical and electrophysiological findings of affected patients in a Vietnamese family with a severe autosomal recessive axonal sensorimotor neuropathy, due to a duplication of 5 nucleotides in coding region of the* GDAP1* gene. This novel variant induces a premature stop codon and may contribute to severe loss of function GDAP1.

## 2. Patients and Methods

We studied a Vietnamese family encompassing all three affected children. The proband and her family underwent clinical and electrodiagnostic evaluations. Genomic DNA was extracted from peripheral blood mainly of the proband and family members using a QIAGEN Blood kit according to the manufacturer's instructions and testing for* GDAP1 *variants. The 6 exons of* GDAP1* were amplified by polymerase chain reaction and analyzed by direct sequencing on an ABI3130XL Genetic Analyzer system (Applied Biosystems, USA). Primers for PCR and sequencing reactions were listed in Supplementary Table 1. Some other common CMT genes (*MPZ, GJB1, MFN2, NEFL,* and* PMP22*) have been analyzed as well.

For the new variant in* GDAP1* gene, 50 unrelated healthy Vietnamese control participants were screened. The variant was numbered according to the Human Genome Variation Society (HGVS) nomenclature on the basis of standard reference sequences of mRNA (NM_018972.2) and protein (NP_061845.2).

## 3. Results

### 3.1. Clinical Features

We described symptoms of the proband and her siblings, ranging in age from 3 to 16 years, and their mean onset age was 27 months.

**Figure 1:**
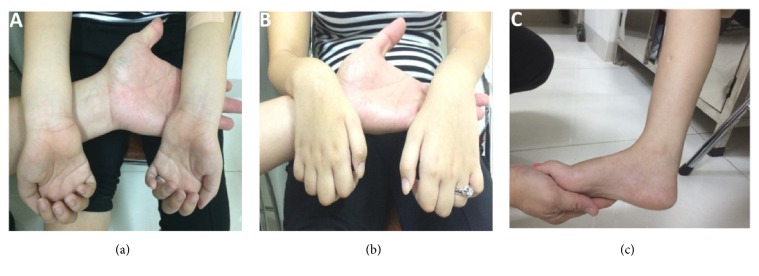
Characteristics of the proband. (a, b) Muscular atrophy in hands; (c) atrophy in the lower legs, pes cavus.

The proband (II-1) had difficulty running starting at 27 months. She was able to independently climb stairs until age 7 and then required ankle foot orthosis at the same year. Due to progressive distal and proximal leg weakness, she needed crutches from age of 10 years. Clinical examination at the age of 16 years showed atrophy of lower legs, lower arms, intrinsic hand muscles, and areflexia. The proximal extremities muscles showed a moderate paresis. There was severe weakness of the lower arm muscles, in particular of the hand muscles. Sensory impairments were not identical in upper and lower extremities: asymmetrical loss of touch and pain sensations with the upper extremities were intact, and vibration was absent in distal. Pes cavus was seen (Figure 1). There was no evidence of hoarseness, vocal cord paresis, diaphragmatic paralysis, or cognitive impairment.

The 12-year-old sister (II-2) and 3-year-old brother (II-3) of the proband were affected, showing the same symptoms as the proband, although to a lesser extent. Both parents (I-1 and I-2) showed no abnormalities on neurological examination and nerve conduction studies (Table 1).

### 3.2. Electrophysiological Findings

Nerve conduction studies of the proband showed no CMAP or sensory nerve amplitude potential (SNAP), which suggested axonal neuropathy. EMG showed signs of de- and reinnervation in the right anterior tibial muscle. In the brother of the proband, CMAP reduces significantly and MNCV was in normal range (Table 2). These data were consistent with a severe sensorimotor axonal polyneuropathy.

### 3.3. Genetic Analysis

**Figure 2:**
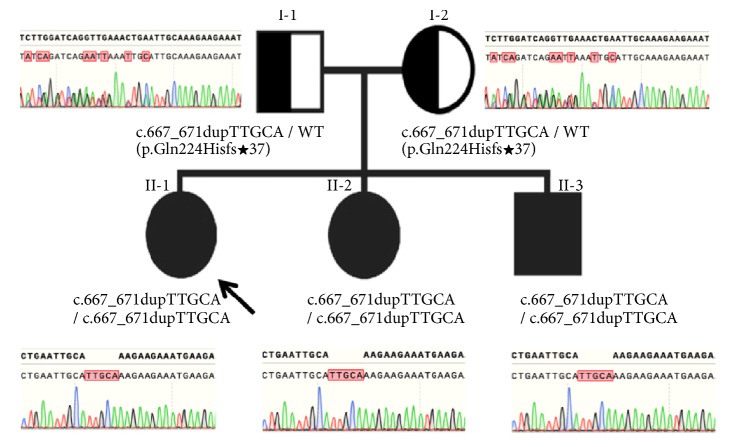
Analysis of* GDAP1* gene (exon 5) in the unaffected father (I-1), unaffected mother (I-2), the proband (II-1), and her siblings (II-2, II-3).

Based on clinical findings, nerve conduction velocities, and family history consistent with autosomal recessive inheritance, a diagnosis of CMT type 4 was suspected. DNA testing revealed a homozygous variation in the* GDAP1 *gene in the proband and her two siblings: c.667_671dup, and a heterozygosity of the c.667_671dup in both parents (Figure 2). The proband and her two siblings carried a frame shift variant p.Gln224Hisfs∗37. However, this alteration was not detected in 50 healthy controls. The Gln224Hisfs∗37 is likely pathogenic variant and not previously reported.

## 4. Discussion

This report described a family with axonal CMT where there was a novel* GDAP1 *variation underlying a typical autosomal recessive phenotype. The affected patients had a disease onset the third year of life with progressive and symmetric hypotrophy and foot deformity. All three of them presented similar disease development and none had mental retardation. The electrodiagnostic results showed a severe axonal neuropathy. It was recorded that the proband's parents are not known to be consanguineous but their origins are from nearby villages in the North area of Vietnam. Both of them have no relevant medical history and no other affected member in multiple generations has been noticed. This inheritance pattern in the pedigree suggests an autosomal recessive disease.

On the basis of a positive familiar history, an axonal sensomotoric neuropathy found in affected patients, early age of onset, and an autosomal recessive pattern of inheritance, alteration of* GDAP1* gene was screened in the family.* GDAP1* gene sequencing showed a homozygous p.G224Hfs∗37 variant, which led us to the confirmation of Charcot-Marie-Tooth disease, type 4A. Both parents are carriers and without risk of developing the disease. Unfortunately, all three children have the homozygous likely pathogenic variant and present similar disease progression.

**Figure 3:**
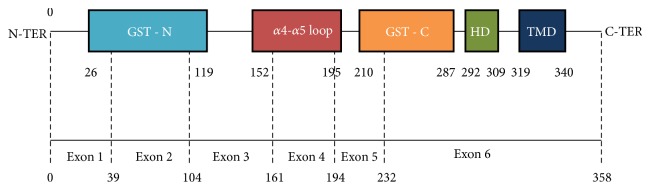
Schematic representation of GDAP1. Glutathione-S transferase (*GST*), hydrophobic domain (*HD*), transmembrane domain (*TMD*), N-terminal (*N-TER*), and C-terminal (*C-TER*). The number indicates the amino acid position in GDAP1 protein.

GDAP1 belongs to the subfamily of glutathione-S-transferase (GST). It is composed of two typical GST domains (GST-N and GST-C), two alpha helical loops (*α*-loop), a C-proximal hydrophobic domain (HD1) crucial for GDAP1-induced mitochondrial and peroxisomal fission, and a C-terminal transmembrane domain (TMD) essential for the correct locating of the GDAP1 protein [8, 18–22] (Figure 3). To date, more than 80 GDAP1 variants have been implicated in the pathogenesis of CMT. Most are missense and nonsense mutations, and some are frame-shift, deletions, mutations generating truncated and nonfunctional proteins, and those altering the splice sites in the* GDAP1* transcripts [15, 23]. Mutations are mostly located in the GST domains of the protein indicating their important roles in the protein function. Most frequent protein consequences are missense followed by frame-shift, nonsense, and splice site mutations. Among the recessively inherited changes, nonsense and frameshift mutations leading to the truncation of the protein produced are very often associated with a most severe phenotype of CMT showing a more rapid course of the disease [15].

GDAP1 mutations are rare in Asian populations, with a reported frequency from 0.6% to 2.37% in Japanese and Chinese CMT patients [24–26]. Conversely, high GDAP1 mutation frequencies are observed in European CMT patients, with reported frequencies approximately 7–14% [27–32].

The p.G224Hfs∗37 variant reported in this study has not been described elsewhere. The mutant protein would thus be shortened to the first 223 amino acids instead of 358 (exon 1 to exon 5), followed by an addition of 37 amino acids and a premature stop codon. We consider this variant very likely pathogenic since GST-C domain has important roles in the protein function, and the premature stop codon inducing a truncated protein may impair some involving processes. Furthermore, DNA-testing revealed no mutations in other common CMT genes and no similar variation is detected in healthy control group. This case illustrates the challenges in elucidating the genetic cause in CMT families as GDAP1-CMT diseases.

## 5. Conclusion

This work broadens the genetic spectrum of CMT associated with GDAP1 mutations with the identification of new G224Hfs∗37 alteration and emphasizes the importance of clinical clues in the diagnosis of inherited neuropathies. It is possible that there are many factors contributing and modulating the GDAP1-CMT and each of the mutations turns the disease in different way, affecting some processes more and some less, thereby explaining such diverse course and severity of CMT disease. More comprehensive information of the genetic background of CMT disease in the Vietnamese is needed in order to describe and understand the effects of mutation on clinical course and prognosis, elucidate the correlation between the genotypes and the clinical phenotypes, and determine the attribution of certain geographical distribution, ethnic background, and areas of high consanguinity.

## Figures and Tables

**Table 1:** Summary of clinical characteristics in proband and other family members.

	II-1	II-2	II-3	I-1	I-2

Gender,	Female,	Female,	Male,	Male,	Female
age at visit	16 yos	12 yos	40 mos	46 yos	45 yos

Age of independent working	13 months	12 months	12 months	-	-

Age of onset	27 mos	27 mos	27 mos	-	-

Muscle weakness	DLL = DUL	DLL > DUL	DLL	No	No

Muscle atrophy	Yes DLL = DUL	Yes DLL > DUL	No	No	No

Tendon reflexes	Absent	Absent	Decrease	Normal	Normal

Sensory loss					
(i) Pinprick	Yes	Yes	Yes	Normal	Normal
(ii) Vibration	Yes	Yes	Yes	Normal	Normal

Deformity	Pes cavus	Pes cavus	No	No	No

Respiratory failure	No	No	No	No	No

DLL: distal lower limbs; DUL: distal upper limbs.

**Table 2:** Electrodiagnostic findings in proband and other family members.

		II-1		II-3		I-1		I-2	
	Normal value	Left	Right	Left	Right	Left	Right	Left	Right

*Median nerve*									
DML (ms)	< 4.4			2.8	3	3.2	3	3.4	3.5
CMAP (mV)	>4	*No response*		*1.6*	*1.6*	7.5	7.3	8.9	12.4
MCV (m/s)	>49			52.4	50	59.5	57.9	55.6	60.6
DSL (ms)	<3.5					2.8	3	3.3	3.1
SNAP (*μ*V)	>20	*No response*		*No response*		34	32	49	42
SCV (m/s)	>50					56.5	54.2	51.5	52

*Ulnar nerve*									
DML (ms)	<3.3			1.75	1.75	2.5	2.5	Not performed	2.7
CMAP (mV)	>6	*No response*		*2.9*	*4.1*	6.2	6.3		5.5
MCV (m/s)	>49			52.8	52.8	61.5	53.4		60.6
DSL (ms)	<3.1					2.7	3.6		2.5
SNAP (*μ*V)	>17	*No response*		*No response*		26	23		42
SCV (m/s)	>50					51	51		57.9

*Posterior Tibial nerve*									
DML (ms)	<5.8					4.2	4.1	3.5	Not performed
CMAP (mV)	>4	*No response*		*No response*		11.9	9.2	11.4	
MCV (m/s)	>41					47.4	49.3	51.5	

*Deep Peroneal nerve*									
DML (ms)	<6.5	*No response*		*No response*		Not performed		4.1	Not performed
CMAP (mV)	>2							2.6	
MCV (m/s)	>44							49.2	

*Superficial peroneal nerve*									
DSL (ms)	<4.4	*No response*		*No response*		4.6	5.2	Not performed	
SNAP (*μ*V)	>6					1.2	0.8		
SCV (m/s)	>40					52.6	47.6		

*Sural nerve*									
DSL (ms)	<4.4	*No response*		*No response*		2.9	2.8	3.4	3.4
SNAP (*μ*V)	>6					12	15	19	21
SCV (m/s)	>40					57.1	52.4	46.2	46.2

*Needle EMG*		De- and reinnervation		Not performed		Normal		Not performed	

DML: distal motor latency, CMAP: compound muscle action potential, MCV: motor conduction velocities, DSL: distal sensory latency, SNAP: compound sensory action potential, SCV: sensory conduction velocities, and EMG: electromyography. Nerve conduction studies were not evaluated in II-2 (proband's sister).

## Data Availability

The data used to support the findings of this study are included within the article.

## Supplementary Materials

Supplementary Materials Supplementary Table 1: primers for PCR amplification and sequencing of GDAP1 gene.
