# Supplementary material for: Novel GDAP1 Mutation in a Vietnamese Family with Charcot-Marie-Tooth Disease
Source: Biomed Res Int. 2019 Apr 24;2019:7132494. doi: 10.1155/2019/7132494 (PMC6507255; doi:10.1155/2019/7132494)
Supplement: Supplementary Materials — Supplementary Table 1: primers for PCR amplification and sequencing of GDAP1 gene. [file 7132494.f1.pdf]

Supplementary table 1. Primers for PCR amplification and sequencing of GDAP1 gene.

GDAP1	G-1F	CGT TCA ATT GCA CCT CCC AG
	G-1R	CAC TGG AGG CGG ATT TCT AGG
	G-2F	GTC GGT AAC ACA GGG AAG CC
	G-2R	CCC AAA CCA CCA TCA TGA CAC
	G-3F	GTC TGA GGT GAG GAG ACA GTG
	G-3R	GTG ACC ATG AGA CAT GCT AGG TC
	G-4F	GCA GAG AAG CAG GGC ATG AG
	G-4R	GGC AAC CAG CAT GCT CAA TAT
	G-5F	CAT AGG AGC TGC CAG TGA GTG
	G-5R	CCT TCA CGT AGA TGC AGA TTG AG
	G-6F	CTC TGA GTG TGG CTG TCA AG
	G-6R	CAA TGA GAC AGG CAG CCA TG
